# Demonstrating a Social Intelligence Analysis Framework for Loneliness: Infodemiology Approach

**DOI:** 10.2196/59861

**Published:** 2026-01-15

**Authors:** Hurmat Ali Shah, Mowafa Househ, Loulwah Alsumait, Altaf Alfarhan

**Affiliations:** 1 The School of Computing and Engineering Bournemouth University Poole United Kingdom; 2 College of Science and Engineering Hamad bin Khalifa University Doha Qatar; 3 Department of Information Science Kuwait University Sabah Alsalem University City Kuwait

**Keywords:** health informatics, loneliness informatics, loneliness theory, health effects, loneliness interventions, ICT-based interventions, social media–based interventions, social media, ICT, lonely, loneliness, social isolation, analysis framework, Twitter, Reddit, behavioral data

## Abstract

**Background:**

Loneliness is a dynamic phenomenon that can be investigated using social media and web data.

**Objective:**

This study aims to introduce a framework for studying loneliness through social media and online data sources. A case study is presented to demonstrate the deployment of this framework and its effectiveness in collecting and analyzing data related to loneliness.

**Methods:**

Our proposed framework involves collecting data from various social media and online sources. We discuss the modalities of analyzing the collected data based on the framework’s defined purpose. The analysis was conducted using tools such as Google Trends, the News application programming interface, X (formerly known as Twitter), Reddit, and other social media platforms. Different types of data were categorized according to the proposed framework to understand and study loneliness comprehensively.

**Results:**

The results demonstrate the effectiveness of our proposed framework in collecting various types of data related to loneliness. Tools such as Google Trends and the News application programming interface provided insights into loneliness trends in specific regions. Social media platforms offered behavioral data on loneliness, which were analyzed using sentiment analysis and social intelligence techniques. Correlations between loneliness and personal-emotional and socioeconomic categories were identified through this analysis.

**Conclusions:**

The framework and tools discussed in this paper complement psychosocial approaches to loneliness, which typically rely on self-report measurements. By incorporating online data perspectives, our framework provides valuable insights into loneliness dynamics, enhancing our understanding of this complex phenomenon.

## Introduction

### Background

Loneliness has global public health consequences. Loneliness not only affects the mental health of people worldwide but also has consequences for physical health [1]. Loneliness is a dynamic phenomenon that is understood from multiple perspectives and disciplines [2]. It can be studied from an information and health informatics perspective. Applying data and data science disciplines to study health in rapidly changing scenarios has led to the development of fields such as infodemiology and infoveillance [3]. There is a demand for a framework that is based on the tools of infodemiology to study loneliness through data sources available online and from social media.

The proposed social intelligence analysis framework for loneliness in this paper has four parts: (1) identifying trends, (2) monitoring the news, (3) exploring the breadth of topics, and (4) finally analyzing the depth of topics. In the first stage, it is important to know how the phenomenon being investigated has a general trend. This stage gives us the overall scope of the topic and its temporal dimensions. When the temporal dimensions are known, we can go to the second stage of analysis, which is to know whether the phenomenon is getting coverage in a specific geographical area. The third stage of the analysis is to use social media data to analyze the correlations and associations of the phenomenon in the geographical area. This part can focus on the breadth or the variety of related topics and correlations. The fourth stage, which can compound the third stage, is to provide details on the topics and correlations of the phenomenon.

This paper aims to provide a demonstration through data collection and data analysis according to the proposed framework. Social media and online sources can help us understand the prevalence of loneliness to devise technology-based and community-oriented strategies to address it. While technology may have resulted in a fragmented and individualized existence, it can also be a great healer. The rise of social media has transformed the way in which we interact with others, offering new opportunities for social connection and communication. Loneliness is a common experience that can have negative effects on mental and physical health, and social media use has been implicated as a potential contributor to loneliness [4]. Governments such as Japan and the United Kingdom have designated positions dedicated to loneliness. In response to rising concerns about social isolation, particularly among older adults and young people, Japan appointed a Minister of Loneliness in 2021 [5]. As can be seen, in addition to piquing the interest of scholars, with the engagement of governments, loneliness has become a component of public health.

### Objectives

The proposed framework uses Google Trends, the News application programming interface (API), and data from X (formerly known as Twitter) and Reddit under the interdisciplinary field of infodemiology. Further studies and discussions in infodemiology can be found in the works by Jia et al [6], Eysenbach [7], and Yu et al [8]. We make a distinction between web and social media sources because social media sources are self-reported and can provide an intimate and personal perspective. By web sources, we mean sources other than social media. Although the main focus of this work was on the United States, country-specific filtering can be used for Google Trends, the News APIs, and X data. The Reddit API does not provide the data for a particular country, so Reddit data on loneliness only includes worldwide posts. This is one of the limitations of this demonstration of the social intelligence analysis framework. Nonetheless, Reddit data can still provide useful insights for the study of loneliness. We used the sentiment intensity analyzer contained in the Natural Language Toolkit (NLTK; Team NLTK) and Valence Aware Dictionary and Sentiment Reasoner (VADER) [9] from the NLTK for various analyses in this study.

This study aims to introduce a framework for studying loneliness through social media and online data sources. The framework is important to understand loneliness using data available online and to complement the theoretical and psychosocial understanding of loneliness.

## Methods

### Overview

Most researchers in the fields of sociology, public health, and psychology have studied loneliness using the University of California, Los Angeles (UCLA), Loneliness Scale [10-12]. The UCLA Loneliness Scale is a measuring instrument developed by Russell [13] at UCLA. It is an essential instrument for assessing subjective perceptions of loneliness. The scale comprises 20 items. The UCLA Loneliness Scale investigates various dimensions of loneliness involving social isolation, relational quality, and self-reliance. Its core domains—social connectedness, relational connectedness, and self-reliance—investigate the availability and depth of social interactions and assess an individual’s capacity to manage loneliness. It has been broadly used in psychological research, specifically in assessing the effects of loneliness on mental health and social behaviors across diverse demographic groups. The UCLA Loneliness Scale is a valued quantitative measure [13]. The proposed framework provides a complete assessment of loneliness, helping to identify, recognize, and theoretically address feelings of isolation, thereby generating discussions about social associations and guiding possible interventions to allay loneliness.

The UCLA Loneliness Scale is a set of questions, whereas our framework collects behavioral information and unstructured text data, in addition to other online data, to understand loneliness. For the sake of brevity, a detailed explanation of the proposed framework is not included in this paper.

### Proposed Framework

The proposed social intelligence analysis framework for studying loneliness leverages a wide range of data sources from across the web and social media, addressing the challenges of extracting meaningful information from the overwhelming volume of available online content. Traditional measures of loneliness, such as the UCLA Loneliness Scale, have long been used in scientific and psychosocial research to assess individuals’ subjective feelings of social isolation, well-being, and connection to others. However, these measures rely heavily on self-reported survey data, which while valuable, only capture loneliness in controlled, specific contexts. In contrast, the proposed framework uses real-time, publicly available online data to offer a more dynamic and expansive perspective on loneliness as it naturally occurs in society. The framework is divided into four key parts: identifying trends, following the news, analyzing the range of topics, and examining the depth of discussion.

In the first stage, identifying trends, Google Trends is used to track the frequency with which people search for loneliness-related terms over time. This tool allows for the analysis of temporal patterns in public interest, offering insights into the external factors—such as societal events, economic downturns, or health crises—that may cause fluctuations in loneliness. For example, spikes in searches for loneliness-related terms might coincide with lockdowns during the COVID-19 pandemic, indicating increased public concern. In addition, Google Trends provides regional data on where these searches originate, helping researchers and policymakers target resources and interventions to the areas most affected by loneliness. Google Trends also offers related search queries, enabling the discovery of connected terms such as “loneliness in older adults” or “loneliness and mental health,” which can guide further research and exploration.

The second stage, following the news, involves analyzing news articles using news APIs, such as the News API, Google News API, and Bing News Search API. News coverage of loneliness reflects broader societal interest and how loneliness is framed and discussed in the media. By examining trends and patterns in news reporting, researchers can gain insights into the causes, consequences, and public perceptions of loneliness. Media coverage often highlights demographic variations, such as the loneliness of older adults or teenagers, and reveals how loneliness is discussed within the context of mental health, social isolation, or public health crises. News stories often feature personal experiences, providing a deeper look into how loneliness affects individuals. In addition, news analysis allows researchers to monitor how public awareness of loneliness evolves and how media framing might influence public attitudes or contribute to the stigma surrounding loneliness.

In the third stage, analyzing the range of topics, the focus shifts to social media platforms, particularly X, where users express their personal feelings and opinions in real time. Through keyword searches and sentiment analysis of X data, researchers can observe the range of experiences and emotional responses associated with loneliness. The short-form, real-time nature of posts on X allows for the collection of self-reported loneliness experiences, capturing personal, emotional, and psychological aspects of the phenomenon. Furthermore, the wide range of topics and hashtags related to loneliness can help researchers understand the broader social, economic, and political factors influencing loneliness, providing a more diverse understanding of the issue.

Finally, in the fourth stage, examining the depth of discussion, platforms such as Reddit provide a more in-depth exploration of loneliness through longer, more detailed posts and discussions. Reddit users often engage in communities, or subreddits, dedicated to specific topics, such as *r/loneliness* or *r/depression*, where they share personal experiences and seek advice. This detailed, often anonymous sharing allows for more honest and comprehensive insights into the complexities of loneliness. The depth of these discussions makes Reddit a valuable tool for uncovering the more nuanced, personal dimensions of loneliness, particularly its emotional and psychological impacts. Reddit’s forum-based structure also allows researchers to track the evolution of discussions over time and identify recurring themes and subtopics, contributing to a deeper understanding of loneliness.

### Demonstrating the Proposed Framework

In the initial implementation of the framework, the aim is to gain an understanding of the underlying patterns associated with the phenomenon under investigation. This initial stage provides a comprehensive view of the topic and its temporal aspects. Once these temporal dimensions are determined, we can proceed to the second stage of analysis, which involves assessing whether the phenomenon is receiving attention within specific geographic regions. The third stage of the analysis entails using social media data to explore the relationships pertaining to the phenomenon within these geographical areas. This stage can either focus on the diversity and the broad spectrum of the associated topics and correlations or delve into specific aspects.

Building on the insights gained in the third stage, the fourth stage involves a more in-depth examination of the topics and correlations associated with the phenomenon. In the following sections, we will explain each of these stages in detail, outlining the tools and methodologies that will be used to facilitate their execution.

This paper aims to demonstrate the social intelligence analysis framework through a case study in which data on loneliness were collected from online data sources. The major contributions of this paper are as follows: (1) demonstrating how data can be collected in an organized way and how to analyze them to gain meaningful insights about the nature of loneliness, (2) demonstrating how different online and social media data sources can provide varied information on the dynamic and changing nature of loneliness, and (3) categorizing the themes and topics associated with loneliness into socioeconomic and personal-emotional or other relevant categories from the data collected and processed through the social intelligence analysis framework.

### Data Collection

As this framework involves four different data sources, the data collection for each data source followed the specifics of the associated API and the rules of the data source. The data sources were Google Trends, the News API, X, and Reddit. First, data from Google Trends were collected for the year 2022. The dashboard of Google Trends allows for searching for a particular country using keywords, as well as searching for a specific year. For the news analysis, we used the News API in Python (Python Software Foundation). The data were collected for the keyword “loneliness” in the United States. On the basis of the data collected in this stage, the analysis could focus exhaustively on specific cities or countries to collect more data about them. However, we did not want to limit the search to one specific country to allow for the collected data to be a proof of concept. The collected data on X were merged based on location, user ID, and post ID to identify posts from the United States. The total number of posts was 100,000. The words “lonely,” “loneliness,” “alone,” “isolated,” and “isolation” were used to retrieve the posts.

The Reddit data collection methodology is relatively straightforward. Reddit is a forum-based social media platform where people post about a topic on a subforum dedicated to it. These subforums are called subreddits. The Reddit API provides access to individual subreddits to download the top posts on a topic, which is determined by the number of upvotes and other parameters of engagement. The posts from the *r/loneliness* subreddit were collected through the Reddit API. The *r/loneliness* subreddit has 13,000 members who can post and comment in this subforum. Reddit has its own algorithm for giving scores (ie, higher visibility to posts), which also contains input from other users in the form of upvoting.

**Figure 1:**
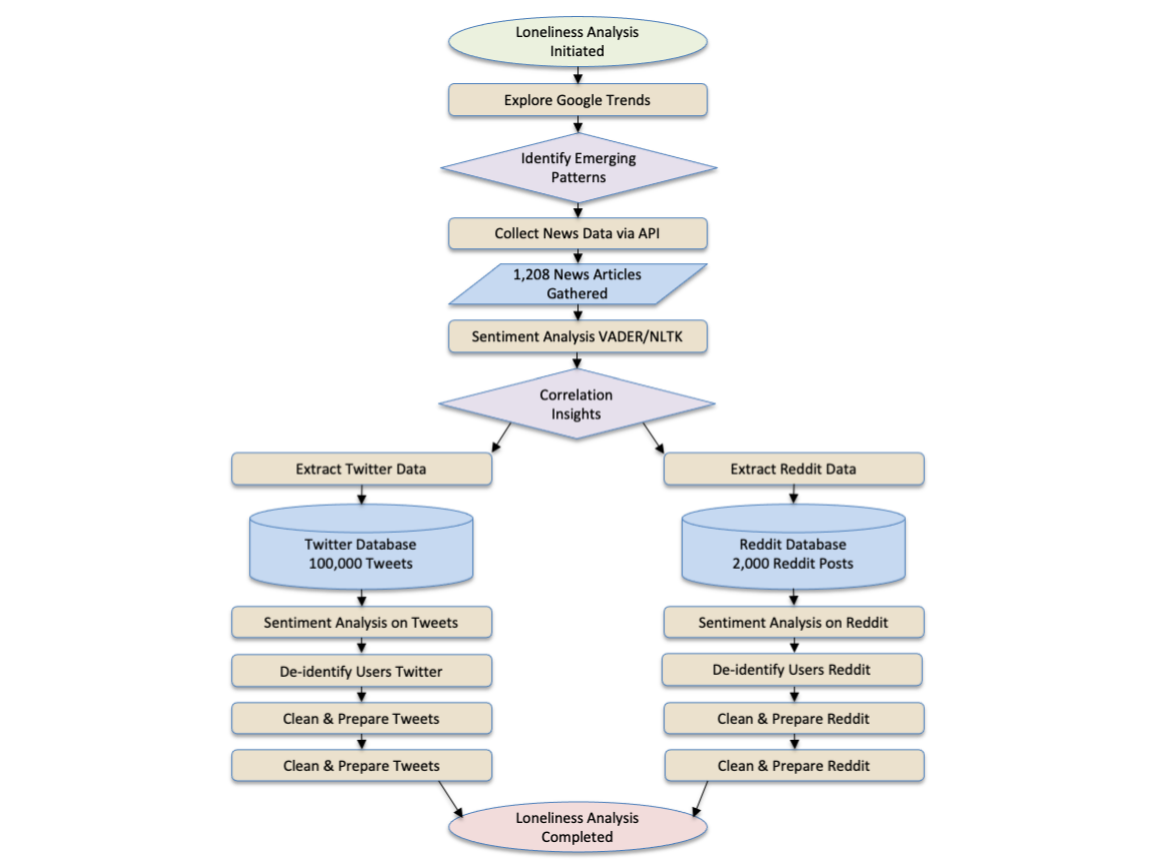
Loneliness framework flowchart. API: application programming interface; NLTK: Natural Language Toolkit; VADER: Valence Aware Dictionary and Sentiment Reasoner.

We collected the top 2000 posts from the *r/loneliness* subreddit with all their comments. The comments varied for each post, both in number and size. It is worth noting that some of the comments were of the same length or even longer than the original posts. Thus, the comments constituted valuable data on loneliness. In total, more than 2000 individual texts were analyzed, which was estimated by multiplying the posts by the average number of comments per post. While some posts did not have comments, the maximum number of comments for a single post was 55. The average number of comments was 4.51, and the total number of comments was 8570. When combined with the posts, this resulted in more than 10,000 unique texts or personal expressions of loneliness from Reddit. We analyzed both the posts and the comments to determine the frequency of occurrence of words to locate the correlations of topics and themes with loneliness. The flowchart in Figure 1 shows the details of the data collection and analysis process used in this study.

### Data Analysis

#### X Data Analysis

We collected a particular number of X posts using keywords related to loneliness. If we reported all the X posts that contained feelings of loneliness, we would not have required a further stage, but the question here is how the expression of loneliness can imply negative consequences, such as mental health problems. In that case, the problem becomes determining the association or correlation between themes (which may represent loneliness) and keywords representing loneliness. For instance, we had to determine the relationship between “hurt,” “sick,” “tired,” and “sleep” and the expression of loneliness. This task is usually carried out by associating lexicon categories with posts including the words “lonely” or “alone.”

**Figure 2:**
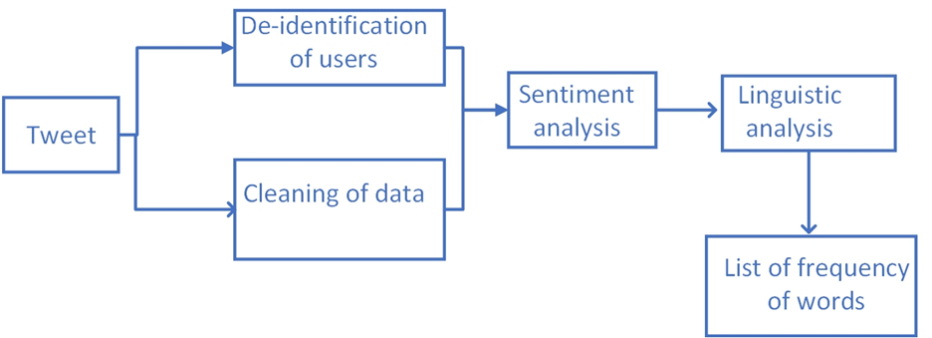
Pipeline for processing Twitter data.

The problem we formulate in this paper is broader in scale. Thus, the limited scale of representative X posts had to be interpreted in a novel way to provide meaningful insight into loneliness. All the posts in the dataset contained keywords representing loneliness. These data could be analyzed to find the association between loneliness and other socioeconomic or personal-emotional categories worldwide or for individual countries. Analyzing these data is important to provide a global picture of the determinants of loneliness and to provide a tool for policymakers to address loneliness in their specific countries. However, “lonely” or “alone” can also be mentioned in a nonnegative way. Using sentiment analysis and manual analysis of the topic and themes of negative posts allowed us to look at the relationship between mentioning keywords representing loneliness and negative emotions, which may ultimately be linked to psycholinguistic features of mental well-being (Figure 2).

Loneliness in the context of mental health is a negative emotion, which is why the sentiment analysis stage is required**―**to find out how loneliness is expressed. For an analysis of loneliness in the context of mental health, we filtered out the X posts in which the expression of loneliness was negative. The collected posts also contained metaphorical uses of “lonely” or “loneliness” that did not pertain to our use of loneliness. Such mentions of loneliness were present in positive- and neutral-sentiment posts. The definition of loneliness in this paper connotes a negative feeling. While loneliness can also be a positive or neutral feeling for some people or at certain times, when it comes to its association with mental health issues, the negative consequences of loneliness must be considered.

We conducted sentiment analysis on both news articles and X data. The news articles were analyzed using the sentiment intensity analyzer contained in the NLTK. The collected posts were stored in a database, and a sentiment analysis was conducted using VADER from the NLTK. VADER is a lexicon and rule-based model for sentiment analysis. The lexicon-based algorithm is constructed using a dictionary that contains a detailed list of sentiment features. In addition, VADER complements the lexicon-based dictionary with grammatical rules that are heuristic in nature and used to determine the polarity of the sentiment. The resulting polarity of the sentiment analysis was used as an indication of loneliness in the dataset. For the sake of brevity, we will not go into the details of using VADER and sentiment analysis. For interested readers, we recommend referring to our previous work [14-16].

#### Reddit Data Analysis

**Figure 3:**
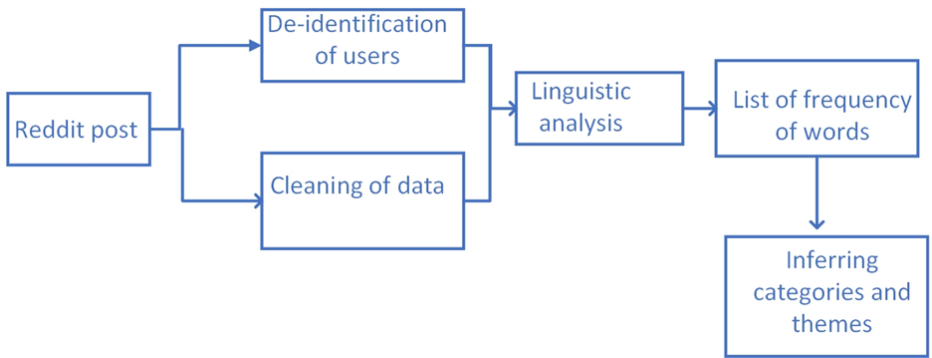
Pipeline to process Reddit data.

Figure 3 shows the pipeline for processing Reddit posts. The difference between Figures 2 and 3 is the absence of sentiment analysis on the Reddit posts. After going over the subreddit *r/loneliness*, we found that the posts were about the emotional expression of loneliness and did not involve metaphorical or non-sequitur uses of “loneliness.” Reddit and its subreddits are characterized by serious engagement on the topics that the subreddits are designed for. Therefore, no sentiment analysis of the Reddit data was deemed important, and the posts were analyzed using the frequency of occurrence of words to find out the themes and topics that were most highly associated with loneliness.

#### Manual Coding and Analysis of X and Reddit Data

The authors cleaned the data before analysis. We ensured that the X posts were deidentified by removing usernames and IDs as part of the data cleaning process. While the data are publicly available, we did not disclose any collected data without first anonymizing them. Sentiment analysis was conducted after cleaning the data, which included removing redundant characters, numbers, special characters, users’ profile IDs, and information such as reposts. For the Reddit data, direct analysis was possible. However, posts from bots or other automated and potentially malicious agents were not filtered out in this study, a limitation that we plan to address in future work by removing such posts before analysis.

We stored X posts with a negative sentiment separately for further analysis, focusing on identifying prominent themes and categories through manual coding. After removing stop words and applying lemmatization to reduce word count, we generated a compact list of word occurrences. This list was manually analyzed to identify larger socioeconomic or emotional-personal categories guided by the literature, although the process remained subjective, relying on the researchers’ judgment. For Reddit data, we followed a similar process, collecting posts and comments, removing stop words, applying lemmatization, and generating a word occurrence list for analysis without conducting sentiment analysis on the data from this platform.

Manual coding and analysis were used to assess expressions of loneliness on X and Reddit objectively. This topic-based categorization was more effective in identifying meaningful similarities and differences. Unlike the n-gram method, which focuses on word co-occurrence, our inductive approach allowed themes to emerge organically, providing a thorough analysis without being constrained by predefined keywords. This method, being quantitative, avoids subjective interpretation, relying instead on the frequency of word occurrences and their classification into relevant categories grounded in existing literature. The detailed analysis method and the use of sentiment analysis for Reddit and X data can be found in our previous work [14-16].

#### News and Google Trends Analysis

The methodology used in this study involved using the News API tool for data analysis. The News API provides programmatic access to a vast collection of news articles from various sources. The data analysis process began by formulating relevant search queries and parameters to retrieve news articles specifically related to loneliness. These parameters included keywords such as “loneliness.” The News API facilitates the retrieval of a significant volume of news articles encompassing different geographical regions and periods. The collected data underwent preprocessing, including cleaning, filtering, and removing duplicate or irrelevant articles. Subsequently, sentiment analysis was used on the news articles. Sentiment analysis for news articles was used for the same reasoning explained previously for the analysis of X posts. These analyses aimed to identify prevalent themes, trends, and sentiments associated with loneliness.

Google Trends provides access to a vast database of search queries and allows for the analysis of search interest over time and across different regions. The data analysis process for Google Trends began by selecting relevant keywords related to loneliness. These keywords were used to retrieve search interest data from Google Trends. The retrieved data were then processed and analyzed to identify temporal patterns, regional variations, and related queries associated with loneliness. The analysis involved examining trend graphs, comparing search interests across different regions, and identifying related topics and queries.

### Ethical Considerations

All data such as usernames, tweets, quotes, etc, in the paper have been deidentified.

## Results

**Figure 4:**
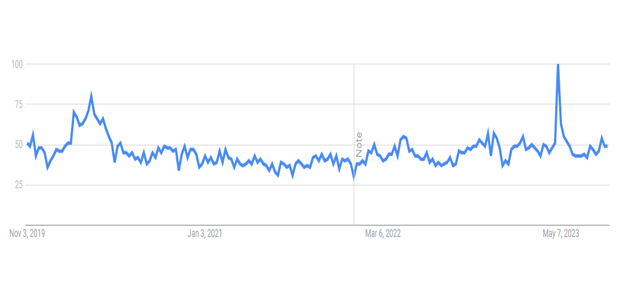
Google Trends chart for the term “loneliness.” The “Note” breakpoint in the graph represents the improvement to Google’s data collection system on January 1, 2022.

As the first stage involved knowing the trends, we carried out a search for the term “loneliness” on Google Trends, shown in Figure 4. We selected a longer period starting before the COVID-19 pandemic, specifically from November 1, 2019, to August 31, 2023. Figure 4 shows a snapshot of the trend graph for “loneliness.” The “Note” breakpoint in the graph represents the improvement to Google’s data collection system on January 1, 2022. The y-axis represents interest over time in the topic. A value of 100 represents peak interest in and popularity of the topic, whereas a value of 50 means that the term had half the popularity. The data points were collected weekly. There was a peak in interest in the topic on May 7, 2023, which did not correspond to a particular event and seems to be an outlier or an anomaly. On the other hand, the interest in the topic was at higher levels during the months of lockdowns related to the COVID-19 pandemic, peaking around the end of March 2020. Overall, the graph shows that the interest in loneliness remained at approximately half the peak levels throughout this period. This shows a sustained interest in the topic. Although the number of searches for the term and its volume are not provided, the popularity rates provide an insight into the trends for loneliness (or any other term) over time.

Google Trends also provides *Related queries* for the topic. In case of the search term “loneliness,” the related queries were “covid loneliness,” “loneliness during covid,” “my loneliness is killing me tiktok,” “is the cure to male loneliness,” and “surgeon general loneliness epidemic.” These terms can express different socioeconomic, personal-emotional, or other phenomena associated with loneliness. If further insight into loneliness is required, these terms can be searched separately, and the results can be compared. Google Trends also provides a tool in which two different topics or queries can be searched.

In the second stage, following the news, we used the News API in Python to retrieve news articles containing mentions of loneliness. In total, we retrieved 956 articles. Table 1 includes a random selection of 25 articles. We carried out a sentiment analysis of the news articles retrieved. An overall negative sentiment score means that the article discussed topics or themes that were negatively associated with either loneliness or broader mental health issues. The news articles with negative sentiment scores can be read for further trend analysis.

**Table 1:** A list of news articles with their sentiment analysis scores.

Article title	Sentiment score
Meet The People Who Listen to Podcasts Crazy-Fast	−0.766
MORABITO: Hillary Clinton Just Gave Away the Left’s playbook for censorship and oppression	0.681
The Connection Cure: 6 Ways to Beat Loneliness	−0.661
Official Trailer for Babak Jalali’s ‘Fremont’	0.077
4 Signs Trauma Has Affected Your Self-Worth	−0.944
Why Historian Jill Lepore Hated Barbie	−0.166
MJ Lenderman Nods to Bob Dylan on New Single “Knockin”	0.700
How Athletic Beer Won Over America	0.215
4 ways simulation training alleviates team burnout	−0.681
Nessa Barrett ON: How to Overcome Loneliness	−0.851
5 Ways Men Can Build Strong Connections	0.971
Gywneth Paltrow saw you from across the bar and wants you to stay with her	−0.296
Album Of The Week: Ratboys The Window	0.250
What is the ‘Joy’ in the Joy of Missing	0.212
Self checkout could be making Americans Lonelier	0.772
Leave it to the dogs (13 Photos)	−0.700
An Easy Way to Reduce Depression And Loneliness	−0.968
Perils of not being attractive or athletic	−0.908
Parents Are Almost as Depressed and Anxious as Teens	−0.900
Bike Happy Hour, listening, and loneliness	0.908
3 Ways Teachers Can Instill Belonging in Students	0.898
Let It Be Sunday, 325!	0.338
How to Overcome Feeling Lonely and Powerless	−0.953
Edinburgh Fringe: The Life and Times of Michael K	0.869

Another analysis that can be carried out on the collected news articles is a list of bigrams in collocations. A collocation is a series of words that co-occur more often than would be determined by chance. In Textbox 1, we collected the bigram collocations (ie, a combination of 2 words that occurred together in the collected news articles). Although the list is a small sample and contains words that may connote difficulties regarding loneliness, collecting bigrams in collocations can provide a wider impression of what themes and topics are discussed in conjunction with loneliness. This, in turn, can point to other directions for exploring the dynamics of loneliness.

**Bigram collocations.**

- “Relationship” and “loneliness”
- “Insomnia” and “symptoms”
- “Loneliness” and “purpose”
- “Depression” and “bitterness”
- “Anxiety” and “disorder”
- “Isolation” and “silence”
- “Alcohol” and “misuse”
- “Anxiety” and “loneliness”
- “Epidemics” and “obesity”
- “Loneliness” and “long”
- “Help” and “loneliness”
- “Insomnia” and “symptoms”

**Figure 5:**
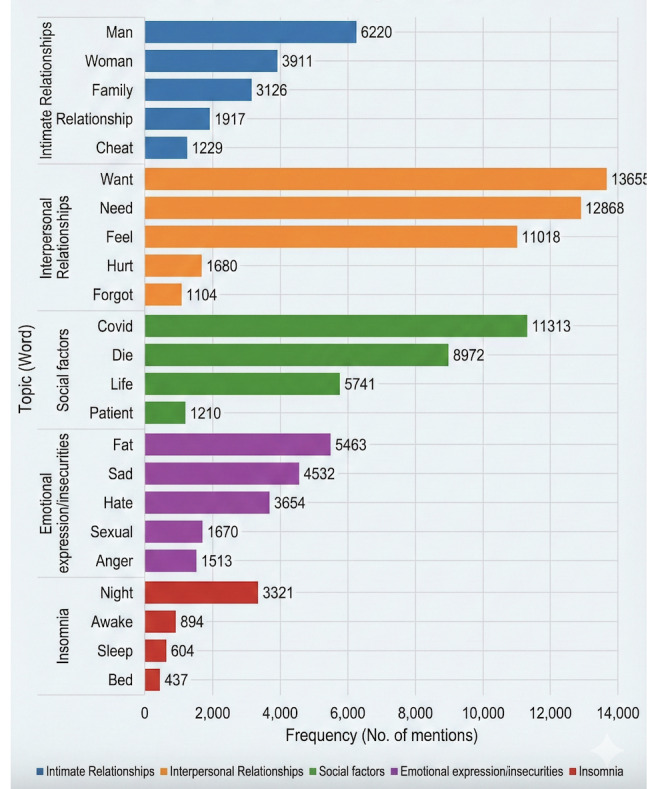
Visualization of highly correlated themes obtained from X posts.

For stage 3, the analysis of the range of topics and topic analysis was conducted on the X posts. Table 2 shows the results of relevant themes and categories from analyzing the word occurrence in posts, whereas Figure 5 shows a visualization of the most dominant themes. We carried out sentiment analysis on 200,000 posts and found that 30.7% (n=61,400) had a negative sentiment. Table 2 breaks down the text of these negative-sentiment posts into the resultant words. Posts containing the keywords mentioned in the Methods section were collected. Sentiment analysis was then carried out. Sentiment analysis differentiates between phrases and topics that carry meaningful information on loneliness and those that use the term in a metaphorical or non-sequitur manner.

**Table 2:** Words highly correlated with mentions of loneliness in X posts. Topics are categorized under a broader thematic area.

Thematic area and topic			Mentions, n
**Intimate relationships**			
	“Cheat”	1229	
	“Man”	6220	
	“Family”	3126	
	“Woman”	3911	
	“Relationship”	1917	
**Interpersonal relationships**			
	“Want”	13,655	
	“Need”	12,868	
	“Feel”	11,018	
	“Hurt”	1680	
	“Forgot”	1104	
**Social factors**			
	“Covid”	11,313	
	“Die”	8972	
	“Life”	5741	
	“Patient”	1210	
**Emotional expressions or insecurities**			
	“Sad”	4532	
	“Hate”	3654	
	“Fat”	5463	
	“Anger”	1513	
	“Sexual”	1670	
**Insomnia**			
	“Night”	3321	
	“Awake”	894	
	“Sleep”	604	
	“Bed”	437	

The results show that most of the X posts containing keywords associated with loneliness from the United States were neutral, which means that they did not meaningfully contribute to the analysis of loneliness. Before conducting the detailed analysis of the posts on loneliness, it was important to identify uses of “loneliness” as a metaphor or non sequitur (ie, those posts that would not add meaningfully to the analysis of negative consequences related to loneliness). Neutrality can also represent the mention of loneliness in descriptive terms.

The basic analysis of the Reddit data for stage 4, examining the depth of the discussions, is provided in Table 3. We collected the top 2000 Reddit posts from the *r/loneliness* subreddit with all their comments. Thus, we analyzed more than 2000 total individual texts. The breakup of the data into words resulted in more than 25,000 words. For the sake of meaningful mentions of topics and brevity, we set a threshold of 50 topics that gave us 411 words to be analyzed. It should be noted that a significant number of these words were language constructs. Only the words that were meaningful in terms of emotions or other expressive qualities were included in the analysis.

**Table 3:** Analysis of frequency of occurrence of words in the Reddit data (N=35,057).

	Words, n (%)
Words occurring >100 times	611 (1.74)
Words occurring >1000 times	78 (0.22)

**Figure 6:**
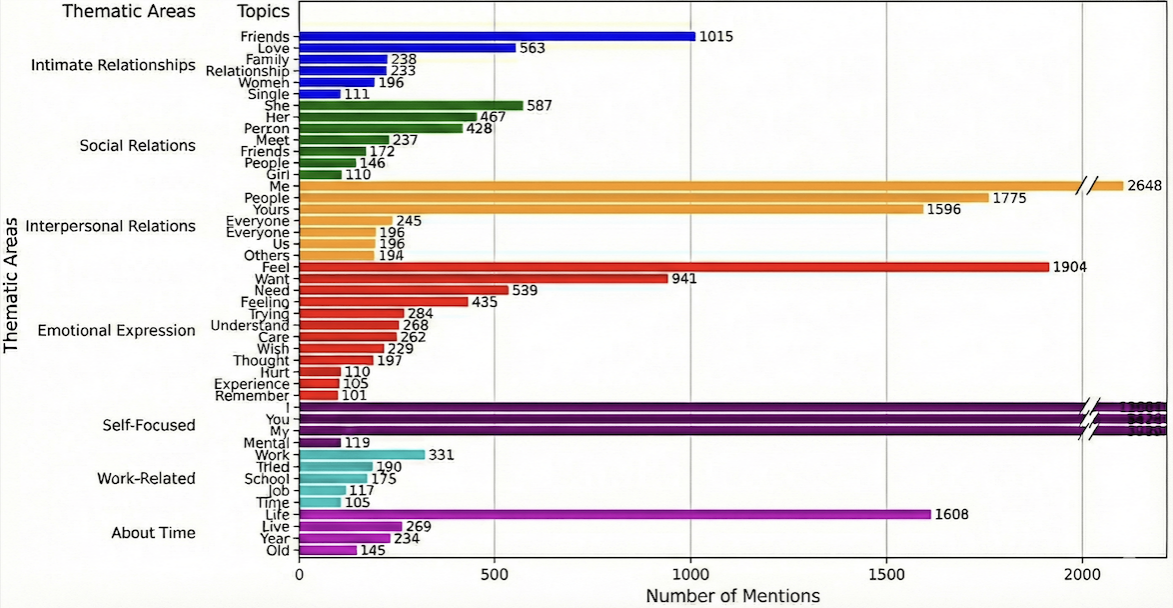
Visualization of highly correlated themes obtained from Reddit posts.

In addition, we want to note that the Reddit posts on loneliness were not specific to the United States. The posts were not divided by country, and the Reddit API does not allow for country-specific downloads. Some methods provide the ability to find the country of the post from the Reddit data, but this involves processes that are beyond the scope of this paper [17]. Table 4 and Figure 6 list and visualize the correlations between themes and loneliness in the *r/loneliness* subreddit. It can be observed from the table that the themes are mostly focused on relations and emotional expression. Because of the longer posts, it is expected that people would have more space to open up and express their feelings. Social media platforms such as Reddit provide spaces where individuals can express their vulnerabilities without facing backlash that can come in the form of social ostracization.

**Table 4:** Correlations of themes with loneliness in the Reddit data. Topics are categorized under a broader thematic area.

Thematic area and topic		Mentions, n
**Intimate relationships**		
	“Love”	563
	“Women”	196
	“Relationship”	233
	“Family”	238
	“Single”	111
	“Friends”	1015
**Social relations**		
	“Friends”	172
	“Girl”	110
	“She”	587
	“Her”	467
	“People”	146
	“Online”	106
	“Meet”	237
	“Person”	428
**Interpersonal relations**		
	“Me”	2648
	“He”	101
	“Yours”	1596
	“Us”	196
	“Everyone”	245
	“People”	1775
	“Others”	194
**Emotional expression**		
	“Thought”	197
	“Hurt”	110
	“Trying”	284
	“Pain”	114
	“Experience”	105
	“Remember”	101
	“Understand”	268
	“Feeling”	435
	“Want”	941
	“Need”	539
	“Feel”	1904
	“Wish”	229
	“Care”	262
**Self-focused**		
	“I”	13,604
	“Mental”	119
	“My”	3989
	“You”	5424
**Work related**		
	“Work”	331
	“Job”	117
	“Tried”	190
	“Time”	105
	“School”	175
**Time related**		
	“Life”	1608
	“Year”	234
	“Live”	269
	“Old”	145

## Discussion

### Principal Findings

This paper demonstrates our social intelligence analysis framework for studying loneliness from online and social media data sources, and presents an overall picture of how a varied topic such as loneliness can benefit from multiple levels of analysis. In this study, we adopted a comprehensive approach, integrating data from Google Trends, news articles, X, and Reddit to examine the multifaceted concept of loneliness within the framework of social intelligence analysis. The demonstration of the social intelligence analysis framework for loneliness revealed interesting patterns, such as in Google Trends, and provided the topics related to mentions of loneliness.

Analysis of Google Trends data exposed intriguing temporal patterns in the public’s interest in loneliness. We observed notable spikes in loneliness-related search queries at various junctures, suggesting that external events, cultural shifts, or seasonal influences may significantly impact the prevalence and perception of loneliness in society. Our examination of news articles provided a broader contextual understanding of loneliness. The sentiment analysis of news articles provided a helpful tool to gather news articles that discuss the negative and health consequences of loneliness.

From a psychological perspective, the increases in the Google Trends graph indicate elevated public interest in loneliness that can be explained as societal reactions to noteworthy or unexpected events [13]. These events, such as the COVID-19 pandemic and the social and physical restrictions that followed, made people feel more emotionally and psychologically alone. Our sentiment analysis of news items revealed that media coverage frequently reflects this elevated awareness. In addition to reporting on these occurrences, the media also influences public opinion by highlighting the psychological results of loneliness, particularly its detrimental consequences on mental health. This combination of social factors and psychological reactions, as observed in media coverage and Google Trends, highlights the multifaceted nature of loneliness [3].

The analysis of X posts and Reddit posts revealed associations between socioeconomic and personal-emotional factors and loneliness. These factors included emotion, sentiment, emojis, and topic modeling. This analysis demonstrated that such factors could help gather evidence and analyze interactions on the topic of loneliness and other related topics. The first factor was emotion, which can serve as a guide in understanding people’s reactions. The second most common factor was relationships. Other thematic areas such as health, work, self-focused topics, and insomnia-related topics indicate the intimate nature of loneliness.

The difference that was observed between the data from X and Reddit (ie, stage 3 and stage 4 of the framework) was in their diversity and extensiveness. In the X data, a range or diversity of topics and themes could be observed. Because of the limited character expression on X, people express their thoughts or opinions in a compact manner; however, through analysis of the terms used and the overall sentiment of the sentences, an association with loneliness can be found. There can be a range of such themes in which there are direct mentions of loneliness in a negative context. On the other hand, Reddit data can be useful for finding the depth of a theme associated with loneliness (ie, what subthemes or topics under a broader category are related to loneliness). These data are important for investigating the possible causes of loneliness. The diversity of the discovered topics and themes from X and the depth of topics that were found on Reddit can be used in complementary ways.

The framework delineated in this paper provides a versatile, multistep approach to analyzing loneliness through online and social media data. Beyond studying loneliness, this framework can be expanded to explore other complex societal issues, such as mental health conditions (eg, anxiety and depression), misinformation, or public reactions to crises. In addition, it can be used for early detection of public health trends or social phenomena by monitoring real-time data. The framework’s capacity for sentiment analysis and topic modeling can offer valuable insights into emotional and psychological responses, which can be applied to develop targeted interventions, inform policies, or enhance public health programs.

The results of this framework reveal the complex, multifaceted nature of loneliness, highlighting its emotional, psychological, and socioeconomic dimensions. These insights can be used in mental health applications by enabling early identification of loneliness trends and allowing for real-time monitoring of at-risk groups. For mental health patient care, these data can be integrated into artificial intelligence–driven tools that personalize interventions, offer resources, or connect patients with support networks. It can also help inform health care providers about socioenvironmental triggers contributing to loneliness. Future research can incorporate more advanced natural language processing tools and extend the use of this framework to cross-cultural studies, improving understanding of how societal factors impact loneliness and other issues across different populations.

The proposed framework can be used in future research endeavors to deepen the understanding of loneliness and its societal implications by providing a systematic approach to analyze diverse and large-scale data from online platforms. By capturing both temporal trends and geographic relevance, researchers can identify key moments and regions where loneliness spikes, enabling a more focused examination of societal or environmental triggers. Expanding the framework to include more detailed demographic information will allow researchers to study how loneliness impacts specific groups, such as older adults or the younger generations, across various cultural contexts. In addition, the framework’s ability to integrate multiple data sources, including social media platforms, news articles, and search trends, offers a more holistic perspective of how loneliness is discussed and experienced at both personal and collective levels. This could lead to a deeper exploration of the role that socioeconomic factors, public health crises, or policy changes play in exacerbating or alleviating loneliness. Furthermore, the sentiment and thematic analysis components can be refined to investigate emotional undercurrents related to loneliness, helping uncover the psychological and emotional dimensions of social isolation.

This framework can support the development of artificial intelligence–driven tools for real-time monitoring and intervention, ultimately informing policy and community-based solutions to address loneliness more effectively. The proposed framework could be adapted to investigate various other societal and public health issues that are influenced by dynamic social and environmental factors. For instance, mental health conditions such as anxiety and depression, which often correlate with loneliness, could be explored by tracking online discourse, sentiment, and search patterns. The framework could also be applied to study the societal impacts of major events such as pandemics, economic downturns, or political crises, where real-time social media analysis could provide insights into public emotions, coping mechanisms, and socioeconomic concerns. In addition, issues such as misinformation, public perceptions of health interventions, or even social phenomena such as digital addiction or climate anxiety could be investigated. By analyzing data from different online platforms, researchers can gain a more comprehensive understanding of public reactions and trends related to these complex, evolving issues.

### Limitations

While our research yielded valuable insights into loneliness using an innovative approach, there are also some limitations. First, our reliance on digital data sources such as X and Reddit may introduce biases. These platforms primarily represent individuals comfortable with sharing their experiences online, potentially excluding those who are less active or lack internet access. This is a limitation of the proposed framework that can be overcome through in-depth interviews or surveys to provide a more holistic understanding of individuals’ emotions, motivations, and coping mechanisms.

In addition, the study’s temporal analysis of Google Trends data lacks causality. While we identified spikes in search queries, determining the specific reasons behind these fluctuations requires further investigation. Furthermore, the focus of the study on English-language data may not fully capture the global diversity of loneliness experiences, potentially limiting the generalizability of our findings. Another limitation lies in the demonstration itself, which relied on data from Reddit, in which the country cannot be specified. For a nuanced understanding of data from Reddit, the data first need to be categorized by region.

### Conclusions

In this paper, we introduced a comprehensive framework for analyzing loneliness through the lens of social intelligence analysis. The framework uses data from diverse online sources, including search engines, news articles, X, and forum websites. This paper provides a demonstration of our proposed framework and reveals correlations between loneliness and online news and posts through sentiment analysis. We provided details on how data can be collected and analyzed according to the umbrella of our proposed framework for studying loneliness through social media and online data. In addition, sentiment analysis of news articles sheds light on the negative health consequences of loneliness, whereas the analysis of X posts and Reddit posts revealed associations between loneliness and various socioeconomic and personal-emotional factors.

Despite the framework limitations, our study provides valuable insights into the multifaceted nature of loneliness through the demonstration of our proposed framework. This study can be used in future research endeavors that can further deepen our understanding of loneliness and its societal implications.

## Abbreviations

API: application programming interface
NLTK: Natural Language Toolkit
UCLA: University of California, Los Angeles
VADER: Valence Aware Dictionary and Sentiment Reasoner

## References

[1] Hards E, Loades ME, Higson-Sweeney N, Shafran R, Serafimova T, Brigden A, Reynolds S, Crawley E, Chatburn E, Linney C, McManus M, Borwick C (2022). Loneliness and mental health in children and adolescents with pre-existing mental health problems: A rapid systematic review. Br J Clin Psychol.

[2] Shah SG, Nogueras D, van Woerden HC, Kiparoglou V (2021). Evaluation of the effectiveness of digital technology interventions to reduce loneliness in older adults: systematic review and meta-analysis. J Med Internet Res.

[3] Al-Garadi MA, Khan MS, Varathan KD, Mujtaba G, Al-Kabsi AM (2016). Using online social networks to track a pandemic: a systematic review. J Biomed Inform.

[4] Peytrignet S, Garforth-Bles S, Keohane K (2020). Loneliness monetisation report: analysis for the Department for Digital, Culture, Media and Sport. GOV.UK.

[5] Ryall J (2021). Japan: 'Minister of loneliness' tackles mental health crisis. DW.

[6] Jia Q, Guo Y, Wang G, Barnes SJ (2020). Big data analytics in the fight against major public health incidents (including COVID-19): a conceptual framework. Int J Environ Res Public Health.

[7] Eysenbach G (2009). Infodemiology and infoveillance: framework for an emerging set of public health informatics methods to analyze search, communication and publication behavior on the internet. J Med Internet Res.

[8] Yu Z, Yang Xi, Dang C, Wu S, Adekkanattu P, Pathak J, George TJ, Hogan WR, Guo Y, Bian J, Wu Y (2021). A study of social and behavioral determinants of health in lung cancer patients using transformers-based natural language processing models. AMIA Annu Symp Proc.

[9] Hutto C, Gilbert E (2014). VADER: a parsimonious rule-based model for sentiment analysis of social media text. Proc Int AAAI Conf Web Soc Media.

[10] Russell D, Peplau LA, Ferguson ML (1978). Developing a measure of loneliness. J Pers Assess.

[11] Wongpakaran N, Wongpakaran T, Pinyopornpanish M, Simcharoen S, Suradom C, Varnado P, Kuntawong P (2020). Development and validation of a 6-item Revised UCLA Loneliness Scale (RULS-6) using Rasch analysis. Br J Health Psychol.

[12] Hudiyana J, Lincoln TM, Hartanto S, Shadiqi MA, Milla MN, Muluk H, Jaya ES (2022). How universal is a construct of loneliness? Measurement invariance of the UCLA Loneliness Scale in Indonesia, Germany, and the United States. Assessment.

[13] Russell DW (1996). UCLA Loneliness Scale (Version 3): reliability, validity, and factor structure. J Pers Assess.

[14] Shah HA, Househ M (2023). Mapping loneliness through social intelligence analysis: a step towards creating global loneliness map. BMJ Health Care Inform.

[15] Shah HA, Househ M (2024). Mapping loneliness through comparative analysis of USA and India using social intelligence analysis. BMC Public Health.

[16] Shah HA, Househ M (2025). Understanding loneliness through analysis of Twitter and Reddit data: comparative study. Interact J Med Res.

[17] Jagfeld G, Lobban F, Rayson P, Jones SH Understanding who uses Reddit: profiling individuals with a self-reported bipolar disorder diagnosis. ArXiv.

